# Monitoring of hourly carbon dioxide concentration under different land use types in arid ecosystem

**DOI:** 10.1515/biol-2022-0534

**Published:** 2022-12-31

**Authors:** Khalid Guma Biro Turk, Abdulrahman O. Alghannam, Faisal Ibrahim Zeineldin

**Affiliations:** Water Studies Center, King Faisal University, Al-Ahsa, 31982, Saudi Arabia; Department of Agriculture Systems Engineering, College of Agricultural and Food Sciences, King Faisal University, P.O. Box 420, Al-Hassa 31982, Saudi Arabia; Water Studies Center, King Faisal University, Al-Ahsa, 31982, Saudi Arabia

**Keywords:** air pollution, carbon dioxide, land use/land cover

## Abstract

Air pollution is a major factor affecting human life and living quality in arid and semiarid regions. This study was conducted in the Al-Ahsa district in the Eastern part of Saudi Arabia to measure carbon dioxide (CO_2_) concentration over different land-use types. Initially, the study’s land use/land cover (LULC) was classified using the spectral characteristics of Landsat-8 data. Then, sensors were placed in five sites of different LULC types to detect CO_2_, air temperature, and relative humidity. The Friedman test was used to compare CO_2_ concentration among the five sites. Five LULC types were identified over the study area: date palm, cropland, bare land, urban land, and water. The results indicated that CO_2_ concentration showed a maximum mean value of 577 ppm recorded from a site dominated by urban lands. During the peak time of human transportation, a maximum value of 659 ppm was detected. The CO_2_ concentration mean values detected for the other LULC types showed 535, 515, and 484 ppm for the bare land, cropland, and date palm, respectively. This study’s sensors and procedures helped provide information over relatively small areas. However, modelling CO_2_ fluctuations with time for LULC changes might improve management and sustainability.

## 1 Introduction

Air quality, air-condition, and climate are major factors affecting human life and living quality [1]. The atmospheric carbon dioxide (CO_2_) resulting from photosynthesis plays a major role in vegetation growth and is a crucial greenhouse gas (GHG) that contributes to global warming [2]. The carbon balance of arid regions can be significantly affected by environmental stresses and human activities [3,4]. The trend of the world population is to inhabit cities by 2050 [5], which might reflect the importance of decreasing GHG emissions [6]. As a result, the global mean concentration of CO_2_ rose steadily from approximately 280 ppm to a level exceeding 400 ppm in the present [7].

Land use/land cover (LULC) changes due to urban expansion are considered crucial factors affecting CO_2_ emissions [8]. LULC change affects the climate through changes in CO_2_ fluxes between the land and the atmosphere [9] and accounting for approximately 10–15% of the atmospheric increase in CO_2_ concentrations [10,11]. However, the LULC change can modulate the land–atmosphere CO_2_ flux at a regional scale compared to the effects of GHG [12]. The direct and indirect GHG emissions from land-use activities such as livestock farming, manure management, fertiliser use, and paddy rice contribute around 12% of today’s total GHG emissions and about 10% rise in CO_2_ emissions from deforestation [13,14]. Nevertheless, terrestrial ecosystems can play an important role in sequestering atmospheric CO_2_ for mitigating the GHG effect [15,16].

The observation of the land surface temperature (LST) in Shenyang, China, indicated that it was higher in urban and bare lands compared to agricultural and green lands [17]. The impact of the local climate zones (LCZ) on LST was investigated in Shenyang city, China’s urban and rural areas, by Zhao et al. [18]. Their findings showed that the LST of LCZs did not follow a fixed order, as they changed from one month to another depending on the land-use type in the urban and rural areas. Therefore, monitoring CO_2_ concentration in urban areas is essential to measure the CO_2_ emission from cities and estimate its contribution to the regional carbon budget [19]. CO_2_ fluxes in urban areas are affected by the vegetative cover, human activity, and climate factors such as precipitation and temperature [20,21]. However, it is crucial to separate the influences of climatic factors for air pollution and environment management controlling over different land-use systems [22]. In cities, the CO_2_ fluxes are controlled by fuel combustion from vehicles, industries, and buildings rather than biological processes [23,24]. Vegetation cover in urban areas can significantly influence the daily and seasonal patterns of the CO_2_ balances [25].

Climate change and LULC change significantly affect CO_2_ dynamics, and the main drivers are temperature and precipitation [26]. For example, the regional drought due to climate change in the lower Mississippi River Valley affected vegetation productivity, net carbon exchange flux, and atmospheric CO_2_ concentration. Thus, the productivity in the drought area decreases by 23% [27]. The intervention of climate change and LULC changes and their impacts on the CO_2_ process were investigated at a global scale using various climate change models; accordingly, for further reading, interested readers are advised to use the following cited literature [11,28–33].

Monitoring CO_2_ levels is an important research theme in most parts of the world [34]. Therefore, a wide range of CO_2_ sensors was developed using different materials for monitoring CO_2_ concentration. A semiconductor sensor was used for detecting CO_2_ for environmental observations [35]. Also, CO_2_ sensors made of solid electrolytes [36–39], optic fibres [39], laser diodes [7], and non-dispersive infrared (NDIR) [40,41] detectors were utilised for observing CO_2_ emissions.

The atmospheric measurement of the CO_2_ concentration depends widely on NDIR sensors because they are stable and robust against interference by other air components, including pollutants [19]. In addition, the NDIR has excellent durability, which makes it the most popular sensor for measuring atmospheric CO_2_ [41]. The calibrated NDIR sensors can reasonably provide accurate air CO_2_ concentrations [7].

In Saudi Arabia, the increasing population within the urban and sub-urban areas puts pressure on natural resources and increases the hazard of CO_2_ emissions. The study area’s land-use system, located in the Eastern part of Saudi Arabia, showed a significant increase in urban lands during the last three decades [42]. Estimating CO_2_ emissions from the LULC changes is considered uncertain due to the difficulty of assessing this flux from measurements [43]. However, in this study, direct measurements of CO_2_ were made to investigate its flux along with the different LULC systems. Hence, the emergence of different patterns of land-use systems in the region will inevitably affect the amounts of CO_2_ emitted. Also, the land-use system usually effectively represents the spatial distribution of CO_2_ emissions and carbon sinks [44]. These conditions necessitate the importance of CO_2_ observation under different arid lands and zones. Therefore, the main objective of this study was to measure CO_2_ concentration over different land-use types. Also, an attempt was made to analyse the impact of CO_2_ emissions on the arid ecosystem of the study area.

## 2 Materials and methods

### 2.1 Study area

**Figure 1:**
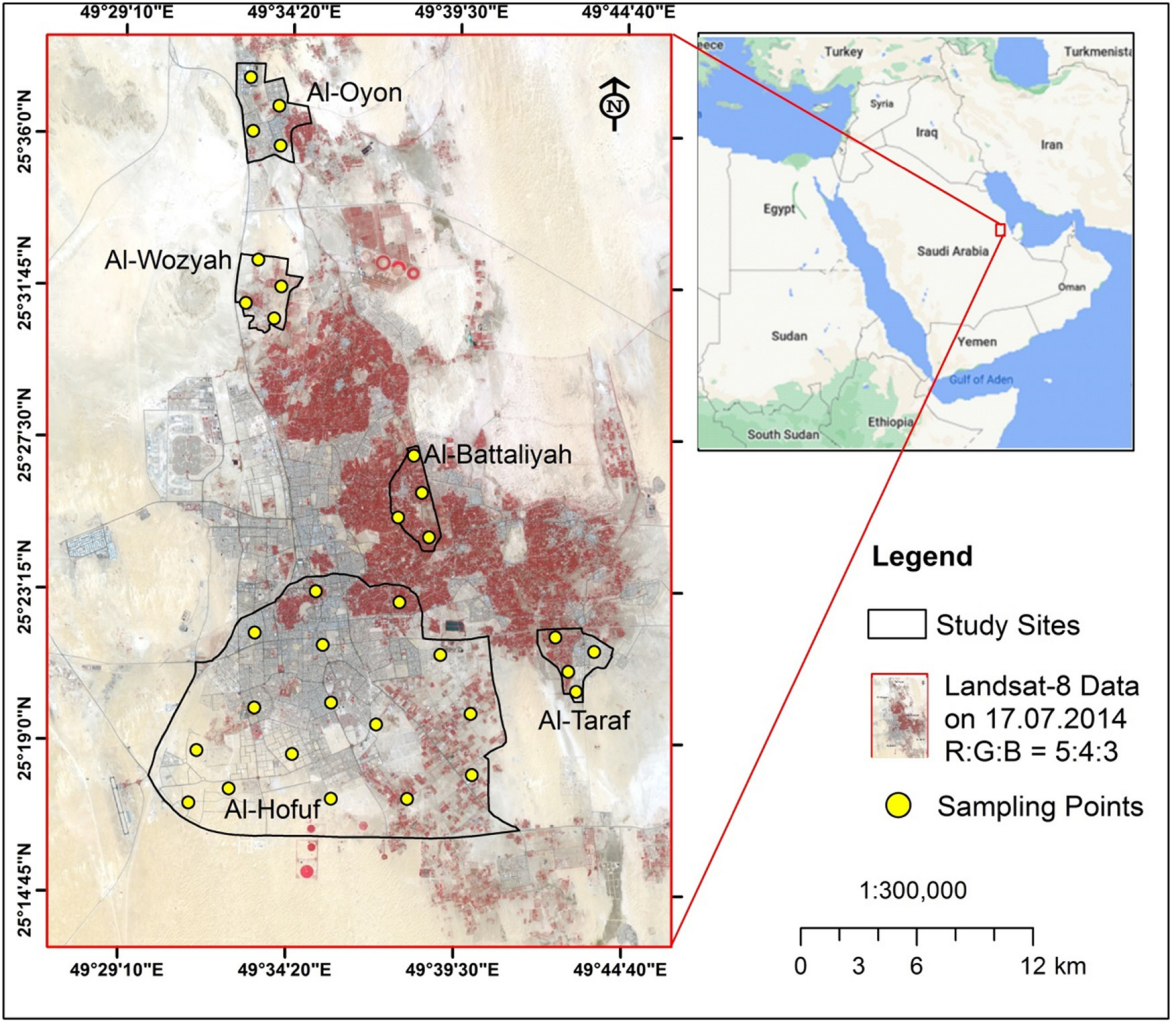
Location of the study sites within Al-Ahsa Zone. The background of the study sites is a Landsat-8 image acquired on 17 July 2014.

The study was carried out in five sites in the Al-Ahsa District located in the Eastern part of Saudi Arabia (Figure 1). The area has a population of 1.1 million (https://www.stats.gov.sa/) and is dominated by a hot, desert climate [45]. The seasonal average temperatures might reach 45°C in summer and 5°C in winter. The rainfall is present only in winter, with less than 250 mm per annum [46]. The land-use system in the study area is predominated by agricultural activities that include date palm plantation and cropping of rice and vegetables. The study sites have different land-use types, areas, and elevations, although they are located in the same climatic zone (Table 1).

**Table 1:** General characteristics of the five study sites and sampling time

Site	Latitude	Longitude	Area (km^2^)	Elevation (m)	Start day and time	End day and time	Total hours (days)
Al-Oyon	25.61000	49.55700	13	112	01 July 2014 06:00:00 AM	30 July 2014 23:00:00 PM	714 (30)
Al-Wozyah	25.52900	49.55700	10	130	01 July 2014 06:00:00 AM	30 July 2014 23:00:00 PM	714 (30)
Al-Battaliyah	25.43000	49.63600	9	140	01 July 2014 06:00:00 AM	30 July 2014 23:00:00 PM	714 (30)
Al-Hofuf	25.32900	49.59800	192	155	01 July 2014 06:00:00 AM	30 July 2014 23:00:00 PM	714 (30)
Al-Taraf	25.35700	49.71700	8	125	01 July 2014 06:00:00 AM	30 July 2014 23:00:00 PM	714 (30)

### 2.2 LULC classification

The LULC classification of the study area was conducted using the Landsat-8 image (path/row is 164/042) acquired on 17 July 2014 (Figure 1) from the United States Geological Survey (https://earthexplorer.usgs.gov/). The Landsat-8 image obtained corresponds with the CO_2_ and climate data collected during the summer season of 2014. The image characteristics are shown in Table 2.

**Table 2:** Characteristics of Landsat-8 data used in this study

Sensor	Bands type	Wavelength (µm)	Spatial resolution (m)
Operational Land Imager and Thermal Infrared Sensor	Band 1, Coastal aerosol	0.43–0.45	30
	Band 2, Blue	0.45–0.51	30
	Band 3, Green	0.53–0.59	30
	Band 4, Red	0.64–0.67	30
	Band 5, Near infrared	0.85–0.88	30
	Band 6, Short-wave infrared 1	1.57–1.65	30
	Band 6, Short-wave infrared 2	2.11–2.29	30
	Band 8, Panchromatic	0.50–0.68	15
	Band 9, Cirrus	1.36–1.38	30
	Band 10, Thermal infrared 1	10.60–11.19	100
	Band 10, Thermal infrared 2	11.50–12.51	100
**Dataset attribute**		**Attribute value**	
Land Cloud Cover		0.00	
Scene Cloud Cover L1		0.00	
Geometric RMSE Model		7.278	
Geometric RMSE Model X		4.239	
Geometric RMSE Model Y		5.916	

In order to identify the LULC within the study area, a field survey was conducted using a Global Positioning System instrument to obtain accurate location point data for each LULC class included in the classification process. The total number of the reference ground control points (GCPs) collected during the field survey was 110. The supervised maximum likelihood classification (MLC) technique was applied to classify the image. MLC is valid and widely used in remote sensing for image classification [47–49]. For image accuracy assessment, 30% of the collected GCPs was used to validate the classification results. In addition, visual interpretation of the unclassified satellite image, Google Earth maps, and field observations were used to verify the LULC maps. The stratified random sampling method was adopted during the image classification to reduce bias [50]. The overall accuracy, user’s and producer’s accuracy, the Kappa statistic, and conditional Kappa were derived from the classification error matrices [51].

### 2.3 CO_2_ and climate parameter measurements

The commercial GE Telaire 7001 carbon dioxide sensor measured CO_2_ and temperature. The GE Telaire 7001 sensor was operating using the dual-beam NDIR technology, and it was connected to an analogue input on a HOBO H22 data logger. The sensor specifications are shown in Table 3, and it has independent CO_2_ and temperature readings. The sensor has been factory calibrated and should be recalibrated once every 12 months using either a zero concentration gas or a gas with a specified concentration of CO_2_ [52]. The sensors were distributed along with the study sites based on the area covered by each side. Accordingly, four sensors were fixed at each site in Al-Oyon, Al-Wozyah, Al-Battaliyah, and Al-Taraf, and a total number of 16 sensors were fixed in Al-Hofuf (Figure 1). The temperature (*T*) and relative humidity (RH) sensors were distributed following the same order as the CO_2_ sensors. The sensors were distributed along the study site to assure data accuracy and fairness. In addition, the average sensor data from each site read the hourly CO_2_, *T* and RH were collected to represent each site fairly. The wind speed data were collected during July 2014 from a local meteorological station in the study area.

**Table 3:** Specifications of the GE Telaire 7001 CO_2_ and temperature monitor

Standards	CO_2_ channel	Temperature channel
Measurement range	0–10,000 ppm display	0–50°C display
Display resolution	±1 ppm	0.1°C
Accuracy	±50 ppm or ±5% for reading up to 5,000 ppm	±1°C
Response time	60 s for 90% of step change	20–30 min
Sample method	Diffusion or flow through (50–100 mL/min)	
Power requirement	100 mA peak, 20 mA average from 6 V	
Operating conditions	0–50°C, 0–95% RH, non-condensing	

### 2.4 Data analysis

The Friedman test was used to compare CO_2_ concentration in the five sites. The Friedman test is a non-parametric analysis used to test the significance for more than two groups; it tests the null hypothesis [53]. Thus, Friedman’s test determines whether the rank totals for each treatment differ significantly from the values expected by chance [54]. The computational formula for the Friedman test [54,55] is

(1) {X}_{r}^{2}=\hspace{0em}\frac{12}{{nK}(K\left+1)}\mathop{\sum }\limits_{j=1}^{k}{R}_{j}^{2}-3{nK}(K+1),]

where *K* is the number of groups (treatments), *n* is the number of subjects, and {R}_{j}] is the sum of the ranked scores in each treatment. Numbers 12 and 3 are constants, not dependent on the number of subjects or experimental conditions. The test statistic {X}_{r}^{2}] is distributed according to the normal {X}_{r}^{2}] distribution with *K*–1 degrees of freedom when the rankings are random. As *n* and *K* increase, the approximation to the {X}_{r}^{2}] distribution improves.

Moreover, Kendall’s *W* test was used to assess the agreement trend among the treatments. Kendall’s *W* is referred to the normalization of the Friedman statistic, and it ranges from 0 to 1. The value “1” refers to the complete agreement between the raters, and “0” refers to the non-complete agreement between raters [56]. Therefore, Kendall’s *W* can be calculated from Friedman’s {X}_{r}^{2}] as follows [57]:

(2) {Ŵ}_{{\rm{kendall}}}=\frac{{X}_{r}^{2}}{(K\left-1)}.]

The Inverse Distance Weighted (IDW) tool of Geostatistical Analyst in the ArcGIS 10.2 software was used to perform data interpolation for the recorded CO_2_ and produce the spatial distribution map [58].

The Statistica [59] and Microsoft Excel 2010 [60] software packages were used to perform the statistical analysis and produce graphs.

## 3 Results and discussion

### 3.1 LULC analysis

**Figure 2:**
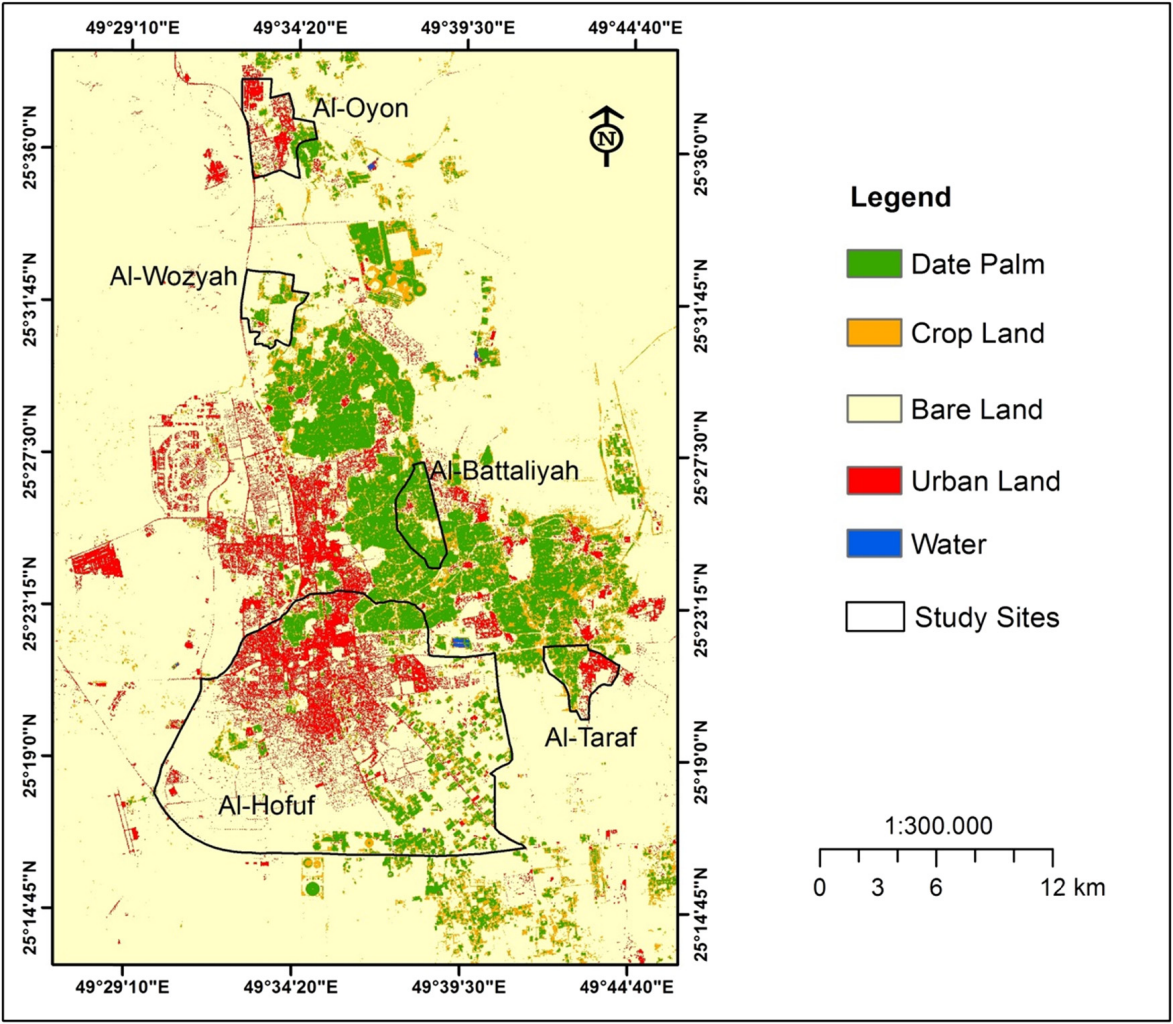
LULC map of the study area on 02 August 2014.

The study area analysis indicated that the existing LULC classes were the date palm, cropland, bare land, urban land, and water (Figure 2). However, within the study sites, the urban land occupied large areas in Al-Hofuf compared to the other sites (Table 4). The date palm orchards were dominant in Al-Battaliyah concerning its relative total occupied area. Bare lands cover about 125 km^2^ in Al-Hofuf, 7 km^2^ for Al-Oyon and Al-Wozyah, and 1.4–3 km^2^ in Al-Battaliyah and Al-Taraf (Table 4). The cropland areas ranged between 1 and 2 km^2^ in all study sites and extended to 12 km^2^ in Al-Hofuf. The LULC map of the study area showed how the different LULC classes were spatially distributed along with the study sites. Thus the LULC types were considered to affect the level of the CO_2_ concentration in a varying way depending on the human activities at each site. Al-Hofuf represents one of the two largest city centres in the Al-Ahsa District. Therefore, urban lands extend in Al-Hofuf, covering large areas. In Saudi Arabia, many residents who lived in the major cities increased from 58% in 1975 to 82% in 2014 [61]. Most urban residents migrated to the cities to seek a modern lifestyle, better employment, and educational opportunities [62]. Also, the built-up area increased by 28.9% during 1990–2014 in Dammam, the capital of the Eastern Region in Saudi Arabia [48].

**Table 4:** Areas of LULC for the different study sites

LULC	Area (km^2^)				
	Al-Oyon	Al-Wozyah	Al-Battaliyah	Al-Hofuf	Al-Taraf
Date palm	1.0	1.4	5.4	19.0	2.0
Cropland	1.0	1.2	2.0	12.0	1.0
Urban land	4.0	0.4	0.2	36.0	2.0
Bare land	7.0	7.0	1.4	125.0	3.0
**Total area (km** ^**2**^)	**13**	**10**	**9**	**192**	**8**

The overall accuracy of the classified LULC map was 90%, with a Kappa statistic of 86% (Table 4). However, the user’s and producer’s accuracies of different LULC classes ranged between 81 and 100%. Also, conditional Kappa coefficients for the different LULC types are shown in Table 5. The user’s accuracy is the probability that a value predicted in a specific class is that class. That means it shows the reality on the ground. In statistical terms, the user’s accuracy measures errors of commission. The producer’s accuracy indicates the proportion of the reference data that are classified correctly for a given class. It corresponds to the statistical concept of errors of omission [49]. Urban lands show low conditional Kappa, user’s and producer’s accuracies compared to the other LULC classes. This can be attributed to the misclassification of some urban land into bare land and cropland. On the other hand, bare lands and water recorded higher accuracies because they have less mixture than the other classes.

**Table 5:** Accuracy assessment of LULC classification

LULC	Classification accuracy (%)		
	User’s	Producer’s	Conditional Kappa
Date palm	92	91	89
Cropland	81	83	78
Bare land	94	93	89
Urban land	79	81	76
Water	100	100	100
**Overall accuracy (%)**	**90**		
**Kappa statistic (%)**	**86**		

**Figure 3:**
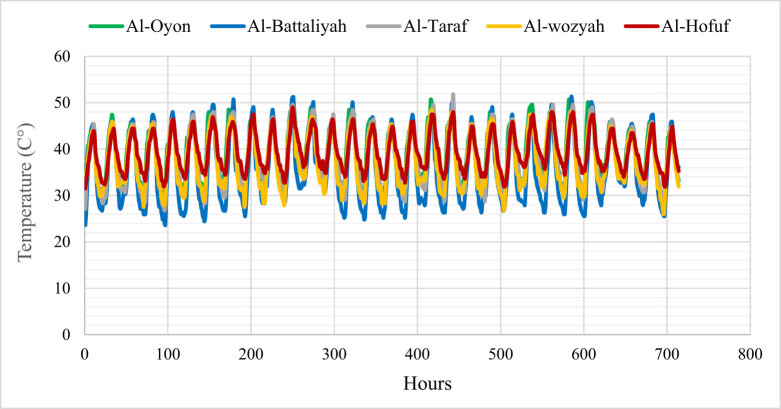
Hourly time series for the *T* levels in the different study sites during July 2014.

**Figure 4:**
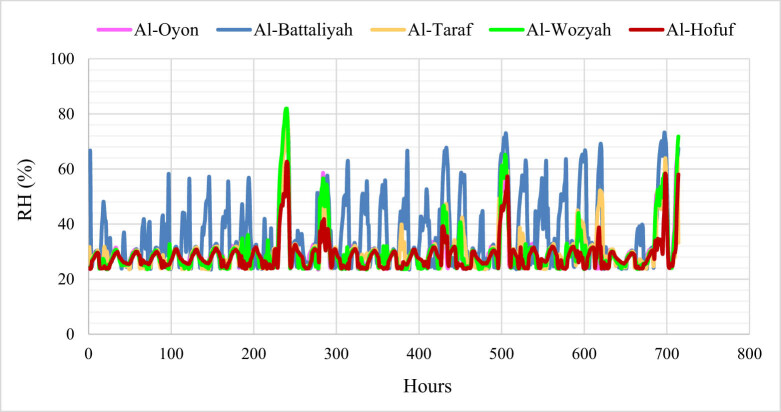
Hourly time series for the RH in the different study sites during July 2014.

**Figure 5:**
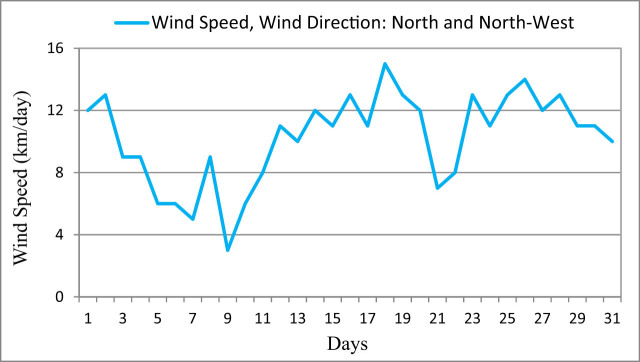
Average daily wind speed over the study area during July 2014.

The hourly recorded *T* patterns are almost similar in all study sites showing a minimum value of 24°C and a maximum value of 52°C (Figure 3). However, the average *T* values ranged between 36 and 39°C; hence sites with large vegetation covers showed relatively low *T* compared to sites dominated by urban and bare lands. The variation of RH along the study sites is clearly shown in Figure 4. The minimum detected RH was 24%, while the maximum was 82%. However, the mean values ranged between 29 and 36%. The low amount of RH can be found during the early morning hours, while the high values occur during the day and most of the evening. Nevertheless, the highest RH value was observed in the site dominated by vegetation cover. The average daily wind speed over the study sites showed a minimum value of 3 km/day and a maximum value of 15 km/day (Figure 5). The dominant wind direction was north to north-west. High wind speed resulted in increasing *T* in urban lands, while the low wind speed decreased the RH in the sites with high vegetation cover.

In urban areas, the physical environmental parameters, including climatic factors, influence pollutant dispersion, which might increase heat island [63]. Therefore, considering the meteorological conditions is important when developing policies to control urban air quality [64].

### 3.2 CO_2_ concentration and levels

**Figure 6:**
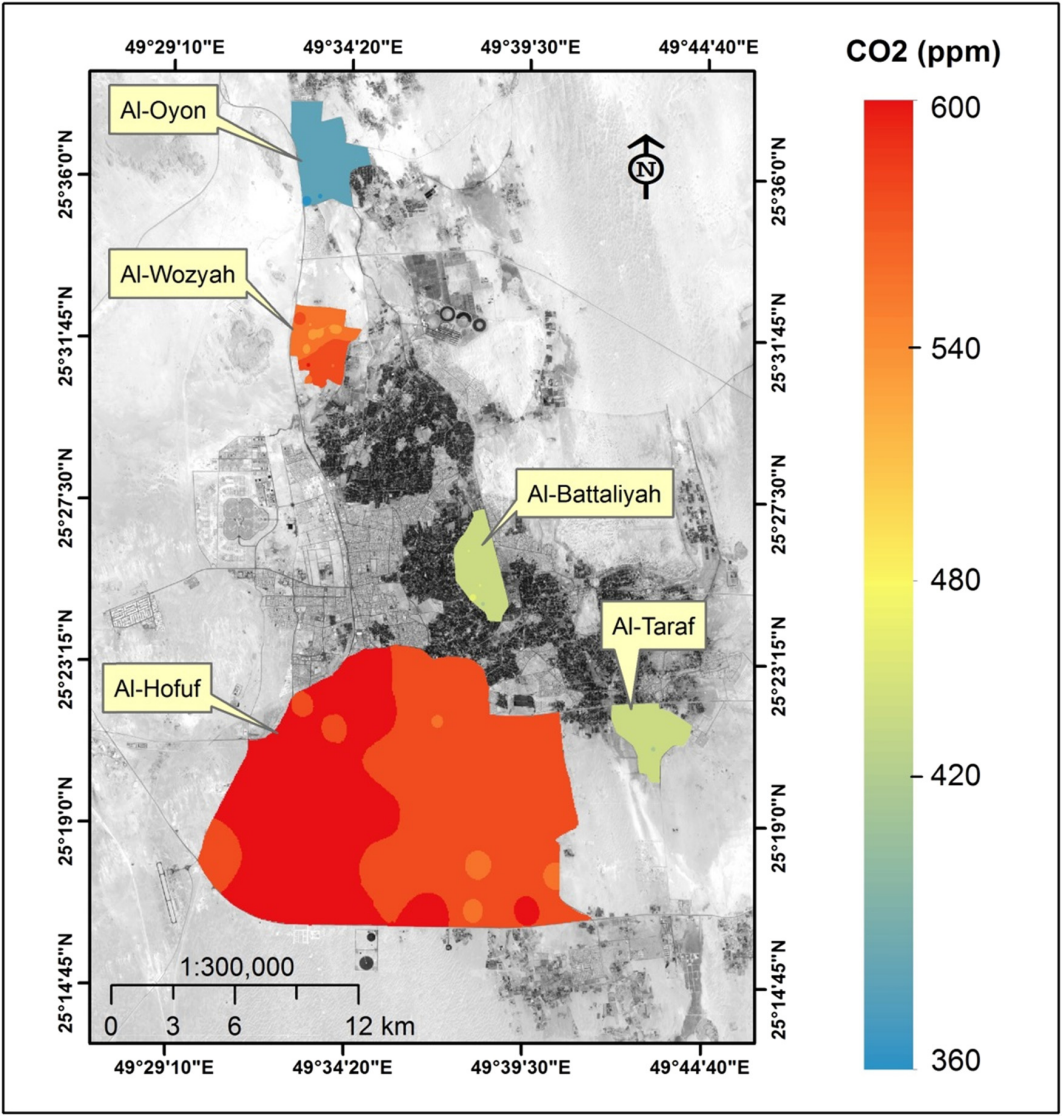
The spatial distribution of CO_2_ levels over the study sites.

**Figure 7:**
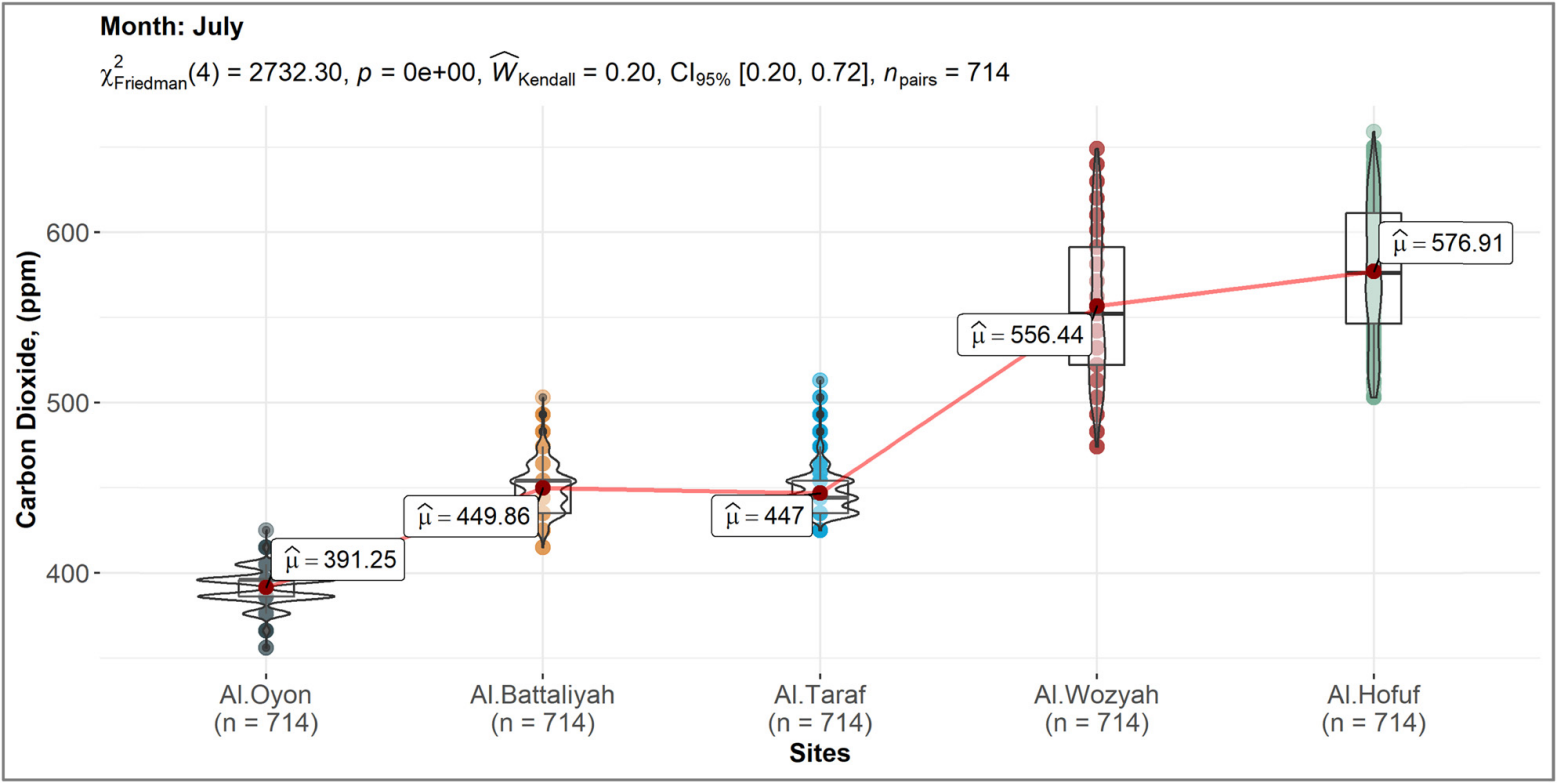
Comparison of the CO_2_ level mean values in the different study sites.

**Figure 8:**
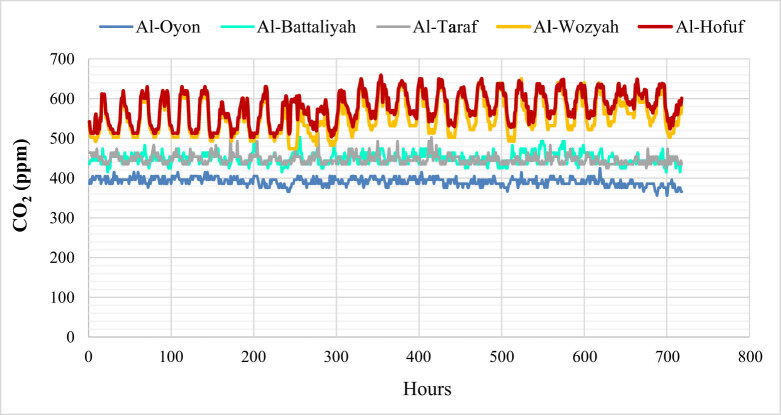
Hourly time series for the CO_2_ concentration in the different study sites.

The spatial distribution of CO_2_ over the different study sites showed high concentration levels in Al-Hofuf and Al-Wozyah, followed by Al-Battaliyah and Al-Taraf. In contrast, low levels were detected in Al-Oyon (Figure 6). The CO_2_ maximum mean value (*μ*^) of 577 ppm was observed in Al-Hofuf, while a minimum mean value of 391 ppm was detected in Al-Oyon (Figure 7). However, the statistical analysis of the CO_2_ levels indicated significant differences (*P* < 0.001) among the study sites based on the Friedman test. Nevertheless, no significant difference (*P* < 0.001) was observed between Al-Battaliyah and Al-Taraf (Table 6). The low value of the {Ŵ}_{{\rm{kendall}}}] test (0.2) indicates that the levels of the CO_2_ differed across the study sites (Figure 7). The hourly time series of the CO_2_ levels were consistent with the spatial distribution of the CO_2_ concentration for the different sites (Figure 8). The time series identified high values of 500–659 ppm and low 356–450 ppm for the CO_2_ concentration among the study sites. In addition, the fluctuations of CO_2_ concentration showed high levels during the peak time of transportation at 7:00–9:00 AM, 1:00–3:00 PM, and 7:00–9:00 PM.

**Table 6:** Pairwise comparison of CO_2_ levels between the different study sites

Friedman coefficient (*X* ^2^) = 2,732		
Degree of freedom (df) = 4		
Probability (*P*) < 0.001		
Sites pair	Statistic	*P*
Al-Oyon**–**Al-Battaliyah	90.14^*^	<0.001
Al-Oyon**–**Al-Taraf	83.22^*^	<0.001
Al-Oyon**–**Al-Wozyah	174.73^*^	<0.001
Al-Oyon–Al-Hofuf	229.36^*^	<0.001
Al-Battaliyah**–**Al-Taraf	6.91^ns^	<0.001
Al-Battaliyah**–**Al-Wozyah	84.60^*^	<0.001
Al-Battaliyah**–**Al-Hofuf	139.23^*^	<0.001
Al-Taraf**–**Al-Wozyah	91.51^*^	<0.001
Al-Taraf**–**Al-Hofuf	146.14^*^	<0.001
Al-Wozyah**–**Al-Hofuf	54.63^*^	<0.001

* = Significant; ns = not significant.

The high concentration levels of the CO_2_ in Al-Hofuf can be attributed to large urban land domination compared to the other sites (Figure 2). The fuel combustion from the cars in Al-Hofuf and the absence of public transport make the situation worse due to the increasing number of private vehicles. In the urban cities of Saudi Arabia, about 92% of the populations depend on private transportation. However, only 32% of these cities are accessible and linked to public transport systems [65]. Also, the energy use in Al-Hofuf is high compared to the suburban areas of the Al-Ahsa district due to the increase in the human population. Therefore, urban areas act as the primary source of CO_2_ to the ambient air [66]. However, the ambient CO_2_ levels could affect the air quality by involving isoprene emission in the vegetated areas [67,68]. Bare lands were the dominant land-use system in Al-Hofuf; therefore, during the summertime in July, this condition might increase the rate of CO_2_ emissions due to the high-temperature degrees. Similar conditions of the vast bare lands can be observed in Al-Wozyah, which also showed high CO_2_ concentrations. Unlike Al-Hofuf, the Al-Oyon site covered large areas of urban and bare lands, but the concentration of CO_2_ in it was low. This is due to the less transportation movement in Al-Oyon, as it is considered a suburban area. Also, palm orchards and farms located in the southeast of Al-Oyon may contribute to reducing CO_2_ emissions. Urbanisation in arid and semiarid regions can significantly impact CO_2_ concentrations and emissions estimated for different LULC types [69,70]. The relatively low CO_2_ concentration in Al-Battaliyah and Al-Taraf might be due to the extensive vegetation covers extended in these sites. Urban vegetation can play a vital role in exchanging CO_2_ concentrations in cities due to plant photosynthesis [40]. Also, urban vegetation is a beneficial planning strategy to control the heat island and improve the energy efficiency of buildings in urban cities [71].

**Figure 9:**
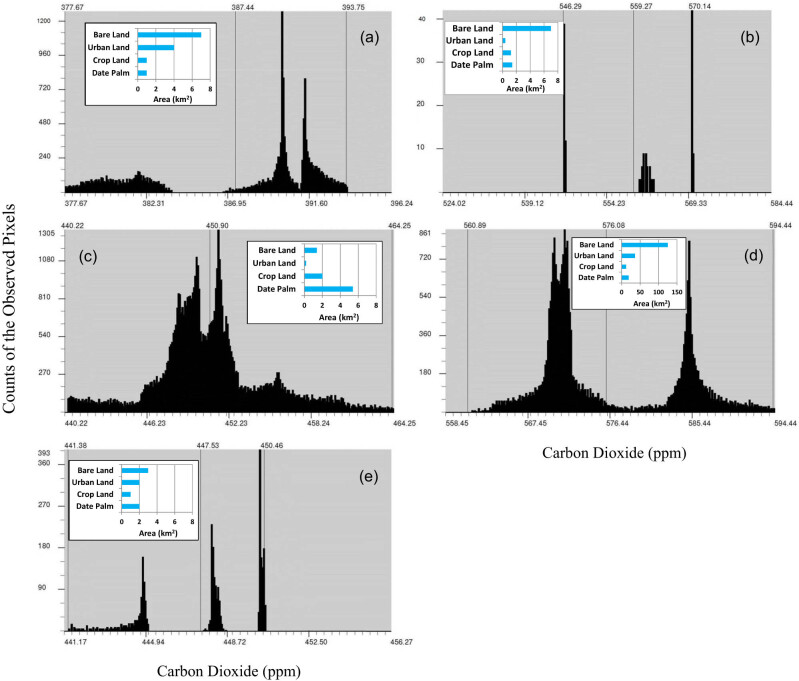
The spatial distribution of CO_2_ concentration over the different LULC types. (a) Al-Oyon, (b) Al-Wozyah, (c) Al-Battaliyah, (d) Al-Hofuf and (e) Al-Taraf.

The spatial distribution of CO_2_ concentration in the study sites shows that its levels vary according to the areas occupied by the different LULC types (Figure 9). In Al-Oyon, the low values of CO_2_ concentration ranging from 377 to 382 ppm occurred in cropland and date palm land-use systems, while the high 386–392 ppm was associated with the bare and urban lands (Figure 9a). Most of the area in Al-Wozyah is covered by bare lands, which shows CO_2_ levels of 559–561 ppm compared to 570 ppm detected over the small area occupied by the urban land (Figure 9b). The high CO_2_ levels resulting from the bare and urban lands in Al-Wozyah have affected the amount of CO_2_ concentration that appeared over the date palm and cropland areas showing CO_2_ levels around 546 ppm. In Al-Battaliyah, the date palm was the dominant LULC system showing CO_2_ concentration levels of 446–452 ppm, followed by the cropland with 440–446 ppm, bare land with 453–458 ppm, and finally urban land with 458–464 ppm (Figure 9c). The domination of bare lands in Al-Hofuf resulted in CO_2_ levels of 560–576 ppm, while the vast areas occupied by the urban lands raised the CO_2_ levels to 580–594 ppm (Figure 9d). The CO_2_ concentration levels in Al-Taraf ranged from 441 to 445 ppm for the date palm and cropland systems, while it ranged between 447 and 449 ppm for the bare land and almost around 450 ppm for the urban land (Figure 9e).

**Figure 10:**
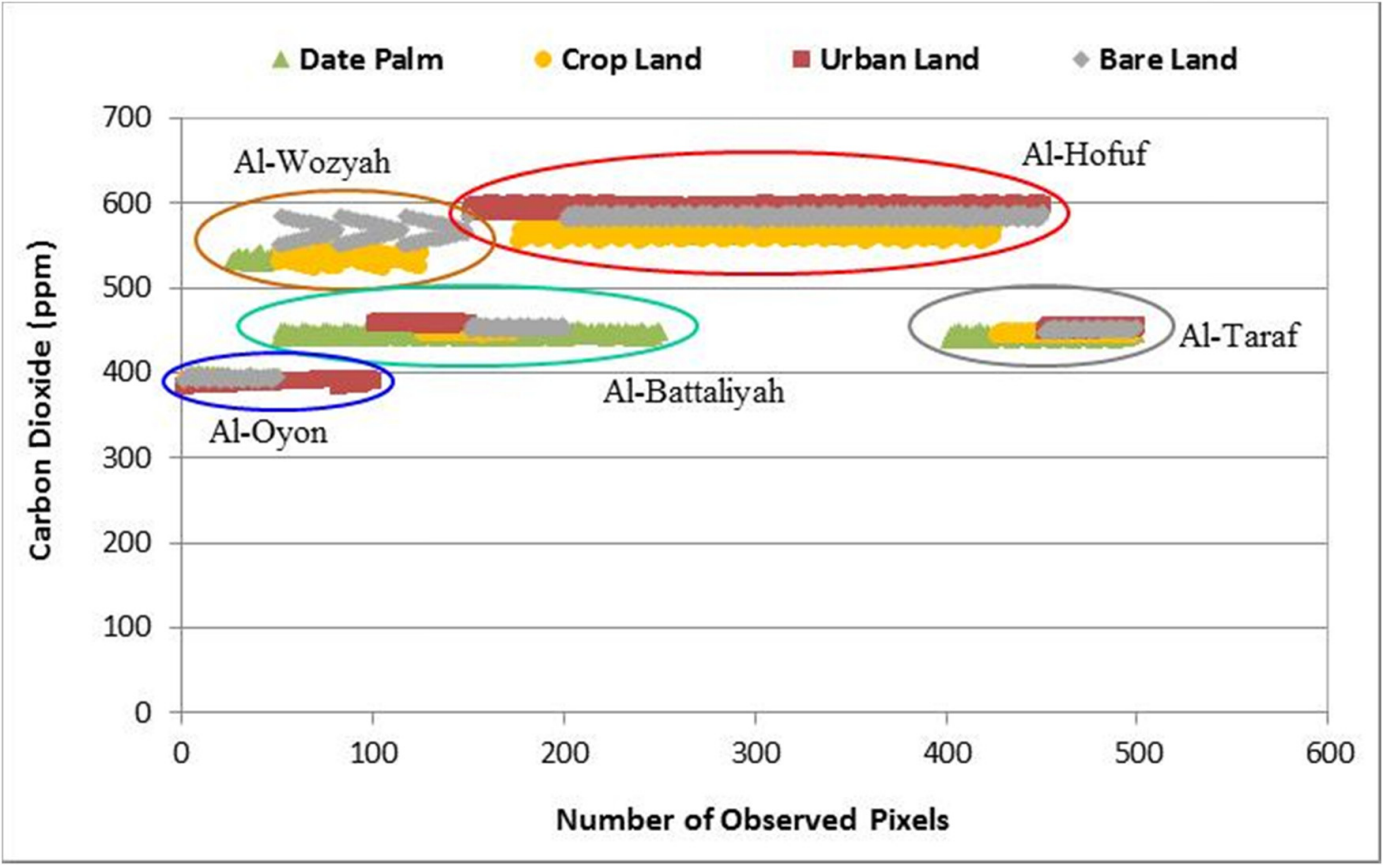
The patterns of CO_2_ for the different LULC types within the study sites.

The CO_2_ concentration mean values detected for the different LULC types in the study area showed it was 560, 535, 515, and 484 ppm for the urban land, bare land, cropland, and date palm, respectively. Thus, the increasing and decreasing of the CO_2_ levels across the different LULC types over the study sites (Figure 10) are mainly attributed to the movement of active winds during the summer, where its speed ranged between 5 and 8 km/h in the north and north-west direction of the study area [72]. Moreover, the urban atmospheric CO_2_ concentrations variation is expected in the Middle East region. For instance, in the Gaza City of Palestine, this variation ranged between 300 and 900 ppm showing higher levels during working days than at the weekend [73]. Therefore, vegetated areas can act as mitigation measure that reduce CO_2_ emissions and lower the air temperature depending on the wind speed and direction from one site to another. Nevertheless, Pataki et al. [74] have indicated the limited role of urban trees in reducing GHG emissions and pollution over vast areas and environmental conditions. However, urban trees are more useful for climate and pollution adaptation approaches than emission reduction. This is because of the limitation in spaces that constrain tree canopies compared to the amount of emissions.

Agriculture and land-use change contribute to about 21% of the global GHG emission. Therefore, to achieve low CO_2_ levels, several measures can be applied. These include re-prioritising land-use systems in urban and rural areas, controlled irrigation, crop diversification, and cover crops [75]. However, land use draws attention to be used as a policy tool for carbon reduction and low-carbon planning [76]. This is because it has changed the Earth’s carbon cycle by influencing the natural carbon sources and sinks like cropland, grassland, and forests [77,78].

Vegetation has crucial environmental functions in urban areas since it removes pollutants [79]. Also, vegetation cover helps in reducing energy consumption in urban areas and hence improves their climate [80]. Therefore, appearing vegetation cover in the urban environment is essential for heat island mitigation in cities [81]. However, CO_2_ was the major driving factor for vegetation cover changes [82]. Moreover, the study area faces a lack of precipitation, which might be one of the main causes of CO_2_ concentration increase [83]. The relationship between urban form and CO_2_ emissions indicated that less complex cities have lower CO_2_ emissions, but dense cities have greater per capita CO_2_ emissions [84]. Also, socioeconomic factors of industrial structure, population density, and economic development level were the main drivers of CO_2_ emissions in urban form areas [85].

## 4 Conclusions

The world faces a continuous increase in atmospheric CO_2_ concentrations due to climate change and the consequences of land-use change. Therefore, continuous monitoring of CO_2_ concentrations in the air at regional and local scales will help drive global efforts to reduce CO_2_ emissions. In addition, site monitoring of CO_2_ will provide the local policymakers with the needed information to formulate policies and strategies that cut down the levels of CO_2_ in the atmosphere.

In this study, the CO_2_ concentrations in the air were monitored for 1 month during the summertime in July. The tested sites covered different LULC types of urban and suburban areas. The hourly recorded data of the CO_2_ showed significant variations between the study sites. For example, a maximum mean value of 577 ppm was detected in a site dominated by urban lands. However, the hourly recorded CO_2_ concentrations showed a maximum value of 659 ppm during the peak time of human transportation and movement. Nevertheless, the patterns of the CO_2_ levels showed significant variations across the different LULC types over the study sites.

The sensors and methodology used in this study provided valuable information about the CO_2_ concentrations and levels over a specific site. However, there is a real challenge in designing a sensor network capable of continuously detecting CO_2_ in large open space areas over a long time. In addition, modelling CO_2_ variation over time due to the LULC changes might help predict the best practices for land-use management that can reduce the CO_2_ levels in the atmosphere. Therefore, adequate management of agricultural lands and reducing CO_2_ emissions and pollution from crop and animal production will help preserve and enhance the air quality for most LULC types dominated in the study area.
